# Surface Display of Multiple Metal-Binding Domains in *Deinococcus radiodurans* Alleviates Cadmium and Lead Toxicity in Rice

**DOI:** 10.3390/ijms252312570

**Published:** 2024-11-22

**Authors:** Liangyan Wang, Yudong Wang, Shang Dai, Binqiang Wang

**Affiliations:** 1 Institute of Biophysics, College of Life Sciences, Zhejiang University, Hangzhou 310029, China; liangyanwang@zju.edu.cn (L.W.); 12207017@zju.edu.cn (Y.W.); 2 State Key Laboratory of Clean Energy Utilization, Zhejiang University, Hangzhou 310029, China

**Keywords:** *Deinococcus radiodurans*, Cd and Pb, rice, outer membrane expression

## Abstract

Cadmium (Cd) and lead (Pb) are the primary hazardous heavy metals that accumulate in crops and pose substantial risks to public health via the food chain. Limiting the migration of these toxic metals from contaminated environments to rice is the most direct and crucial remediation approach. Bioremediation with microorganisms has been extensively utilized for managing heavy metal contamination in the natural environment, and the interplay between microbes and crops is important to alleviate heavy metal stress. Here, we express Lpp-OmpA fused with two metal-binding domains (PbBD and MTT5) in the outer membrane of *Deinococcus radiodurans* to enhance both Cd and Pb adsorption. Our results showed that the recombinant strain LOPM, which displayed an increased tolerance to both Cd and Pb stress, exhibited a 4.9-fold higher Cd adsorption and 3.2-fold higher Pb adsorption compared to wild-type strain R1. After LOPM cells colonized the rice root, Cd content reduced to 47.0% in root and 43.4% in shoot; Pb content reduced to 55.4% in root and 26.9% in shoot, as compared to the plant’s exposure to Cd and Pb. In addition, cells of LOPM strain colonized on rice roots alleviate Cd- and Pb-induced oxidative stress by reducing ROS levels and enhancing antioxidant enzyme activities in rice. This study supplies a promising application of genetic-engineering extremophile bacteria in reducing heavy metal accumulation and toxicity in rice.

## 1. Introduction

Contamination of crops by hazardous heavy metals noticeably impacts food safety and crop productivity. More importantly, it may pose a substantial risk to public health [1,2,3,4]. Cadmium (Cd) and lead (Pb), being metals with high solubility and mobility, could easily be absorbed by plants and have been the main toxic metallic elements that enriched in rice [3,5]. As an important economic crop, rice is the staple food of over half of the world’s population. Daily consumption of rice with heavy metals pollution might be harmful to human health. The accumulation of Cd in the human body can lead to various diseases, such as anaemia, renal failure, and pneumonedema [6,7]. Also, the accumulation of Pb via the food chain usually affects intellectual growth in children [8,9]. Therefore, it is crucial to reduce the absorption and enrichment of cadmium and lead in rice for ensuring the safety of food and public health.

Bioremediation is a promising method for the elimination of toxic heavy metals, owing to its simplicity, economically effective, and environmentally friendly. Many bacteria, including *Bacillus*, *Pseudoalteromonas* sp, and *Rhodobacter sphaeroides* [10,11,12,13], had been applied for removing heavy metal from contaminated soil. Some bacteria have developed resistance strategies to mitigate the damaging effects of heavy metals that accumulate within their cells, including efflux transport, precipitation, thiol-containing compounds, and intracellular sequestration by heavy metal-binding protein.

Metallothionein (MT), a protein rich in cysteine, is capable of sequestering heavy metal ions [14,15]. In *Tetrahymena* spp, 41 MTs have been reported [16]. Among these MTs, MTT5 has been proved efficiently for Cd^2+^ coordination and bioaccumulation [17,18,19]. Heavy metal resistance systems in bacteria are usually regulated by the MerR family, including mercury detoxification (MerR), resistance to cadmium (CadR), zinc (ZntR), lead (PbrR) [20]. The Pb^2+^ responsive regulator PbrR consists of two functional domains: a DNA-binding domain in the N-terminal and a metal-binding domain in the C-terminal, which could specifically bind Pb^2+^ [21,22]. Engineered bacteria expressing proteins that sequester heavy metal have been promising bioremediation applicability. Outer membrane protein OmpA has been used for surface display of function protein, and also applied for heavy metal bioremediation [23,24,25,26,27]. Natural water bodies and soils are often simultaneously accompanied by various heavy metal pollution. However, few studies have investigated surface display of multi-protein in bacteria for multiple heavy metal bioremediation.

The extreme bacterium *Deinococcus radiodurans*, which has been found in soil [28], has unparalleled resistance to various oxidative stresses, including radiation and oxidants [29]. Cd and Pb can generate ROS either directly or indirectly, thereby damaging biomacromolecules like proteins and DNA [1,2,29,30]. Our previous study demonstrated that *Deinococcus radiodurans* could alleviate the toxicity of heavy metal in rice [31], thus engineering *Deinococcus radiodurans* may be a promising way for efficient bioremediation.

Here, we investigated the surface display of multiple metal-binding domains in *Deinococcus radiodurans* that enhance its Cd and Pb tolerance and adsorption. Enhanced root colonization of *Deinococcus radiodurans* recombinant strain LOPM also increased the resistance of rice to Cd and Pb stress and promoted enzymatic antioxidants of rice under Cd and Pb stress. Our results demonstrated that surface display of multiple metal-binding domains in *D. radiodurans* could effectively reduce the accumulation and toxicity of Cd and Pb in rice.

## 2. Results

### 2.1. Surface Display of Multiple Metal-Binding Domains in Deinococcus radiodurans

**Figure 1:**
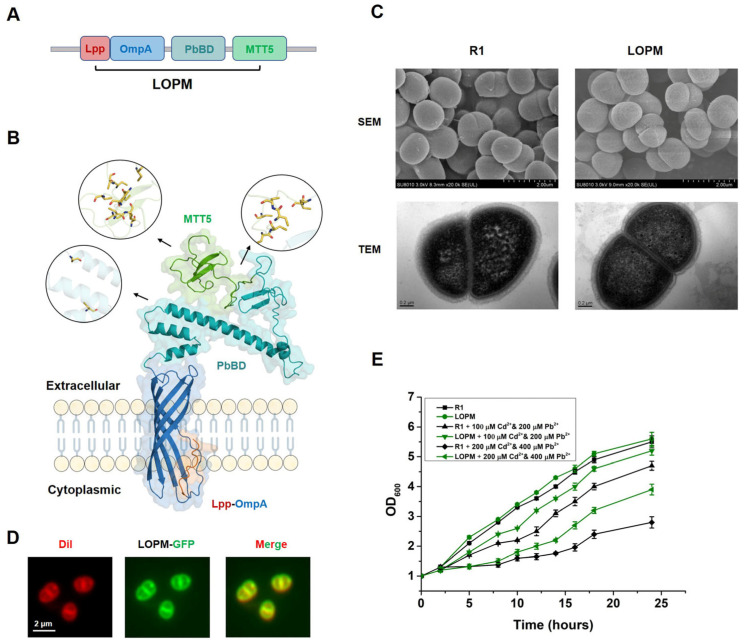
Surface display of multiple metal-binding domains in *Deinococcus radiodurans* by gene fusion expression. (**A**) Schematic diagram of fusion protein module. LOMP stands for the fusion protein composed of Lpp-OmpA, PbBD and MTT5. (**B**) The structure of fusion protein LOPM is predicted by Alphafold 2. The circular area containing reducing cysteine (shown as yellow stick) is the close-up view of the region indicated by the arrow, respectively. (**C**) Scanning electron microscopy (SEM) and transmission electron micrograph (TEM) analyses of *D. raiodurans* wild-type strain R1 and recombinant strain LOPM. (**D**) Localization of LOPM using fluorescence labeling analysis. Scale bars = 2 μm. (**E**) Effect of Cd and Pb on the growth of *D. radiodurans* wild-type strain R1 and recombinant strain LOPM. Bacterial growth was observed under different concentrations of Cd and Pb.

We achieved MTT5 and PbBD expression on the surface of *Deinococcus radiodurans* using Lpp-OmpA signal peptide. As shown in Figure 1A, the whole chimeric protein (LOPM) includes Lpp-OmpA, PbBD and MTT5. Moreover, the structure of chimeric protein LOPM was predicted using AlphaFold 2 (Figure 1B). The results showed that the transmembrane Lpp-OmpA domain consists of a lipoprotein signal sequence (Lpp) and five-stranded antiparallel β-barrel (OmpA). The PbBD domain (showed as cyan color) contains a metal-binding domain with a central linker. The MTT5 domain (shown as green color) with disordered structures contains large amounts of cysteine which participate in metal ion binding (Figure 1B). To determine LOPM cellular localization, the GFP gene was fused to the C-terminal of LOPM; then, the fused protein was expressed in *D. radiodurans* and observed by using fluorescence microscope. The results showed the green fluorescence existed mainly in the cell membrane (stained by Dil), indicating that LOPM was localized in the cell membrane (Figure 1D). By using scanning electron microscopy and transmission electron micrograph analyses, we found that the overexpression of LOPM does not significantly affect the morphology of the cell envelope (Figure 1C). Also, overexpression of LOPM also did not affect the cell growth in normal conditions (Figure 1E). However, the strain LOPM obviously showed a stronger tolerance under Cd and Pb stress (Figure 1E, Figure S1 and Figure S2).

### 2.2. The Recombinant Strain LOPM of D. radiodurans Effectively Absorbs Cadmium and Lead Ions

**Figure 2:**
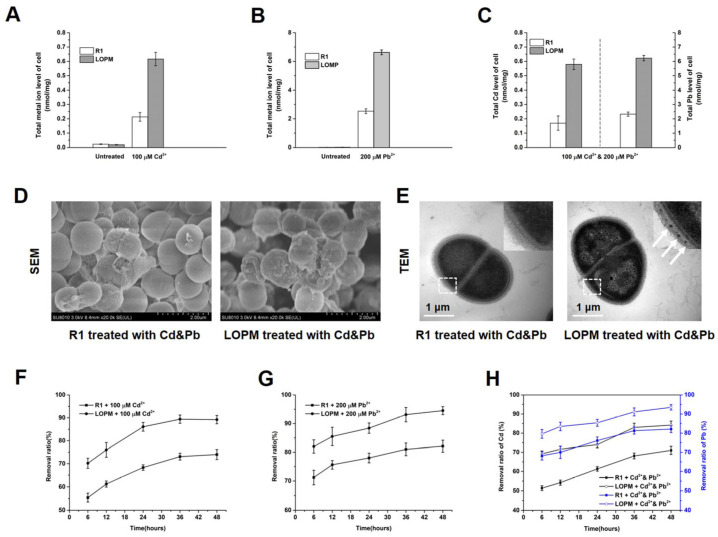
The total amount of Cd and Pb absorbed by the *D. radiodurans* wild-type strain R1 and recombinant strain LOPM. The three different treatments with medium containing 100 μM Cd (**A**), 200 μM Pb (**B**) and 100 μM Cd and 200 μM Pb (**C**), respectively. Cell morphology of *D. raiodurans* wild-type strain R1 and recombinant strain LOPM using SEM (**D**) and TEM (**E**). (**F**–**H**) Cd and Pb removal rates of *D. raiodurans* wild-type strain R1 and recombinant strain LOPM in medium containing 100 μM Cd (**F**), 200 μM Pb (**G**) and 100 μM Cd and 200 μM Pb (**H**), respectively.

Cells from the wild-type strain R1 and the recombinant LOPM strain were suspended in 100 µM CdCl_2_, 200 µM PbCl_2_ and 100 µM CdCl_2_ and 200 µM PbCl_2_ solutions for 2 h, respectively. ICP-MS analysis shows that recombinant strain LOPM and the wild-type strain R1 contain little Cd and Pb in the TGY medium. With the supplementation of Cd or Pb singly, the recombinant strain LOPM showed an enhanced accumulation of Cd and Pb, with levels reaching 2.9 and 2.6 times higher, respectively, than those observed in the wild-type strain (Figure 2A,B). Treating with Cd and Pb simultaneously, the recombinant strain LOPM accumulated 2.6 times more Cd and 2.7 times more Pb than the wild-type strain (Figure 2C). Both SEM and TEM images displayed that the cell envelope of recombinant strain LOPM was covered with small particles after exposure to the mixed heavy metals (Figure 2D,E), whereas almost no obvious particles were found in the cell envelope of the wild-type strain (Figure 2E). After treatment with Cd or Pb singly, recombinant strain LOPM both exhibit a higher removal efficiency than those observed in wild-type strain R1 (Figure 2F,G). After 48h treatment with Cd and Pb, recombinant strain LOPM removed 84.9% Cd and 94.1% Pb from the medium, exceeding the removal rates of the wild-type strain R1 (72.3% for Cd and 82.5% for Pb) (Figure 2H). All these data demonstrated that recombinant strain LOPM had better Cd and Pb absorption efficiency than that of wild-type strain R1, thus it may serve as a suitable strain for the bioaccumulation of Cd and Pb.

### 2.3. D. radioduran Recombinant Strain LOPM Significantly Alleviated Cd and Pb Toxicity in Rice

**Figure 3:**
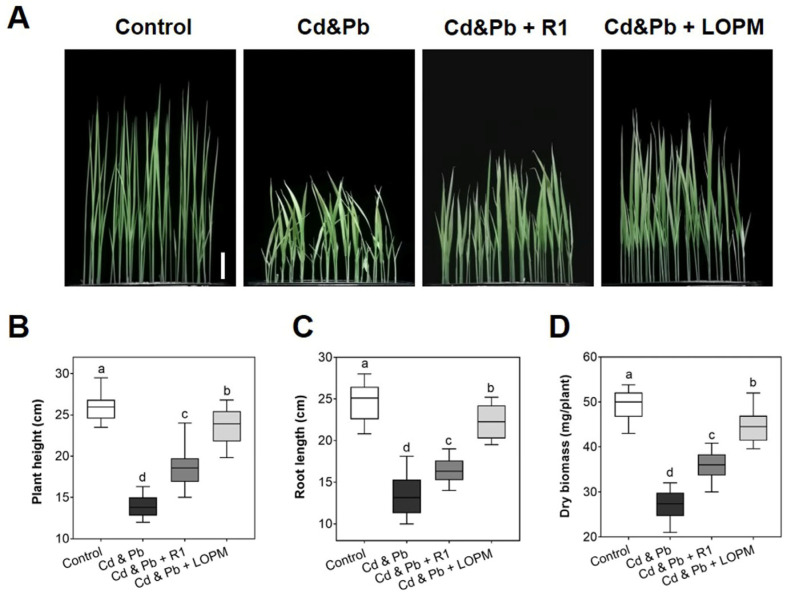
Impact of *D. radiodurans* supplementation on rice seedlings under cadmium and lead stress (Cd and Pb). Scale bar = 5 cm. Data were shown in mean ± SD with 20 (**A**–**C**) and 3 (**D**) repeats. Different letters indicate the significant differences with *p* ˂ 0.05. Medium compositions: Control: 0 μM CdCl_2_ and 0 μM PbCl_2_; Cd and Pb: 100 μM CdCl_2_ + 200 μM PbCl_2_; Cd and Pb + R1: 100 μM CdCl_2_, 200 μM PbCl_2_ and *D. radiodurans* wild-type strain R1; Cd and Pb + LOMP: 100 μM CdCl_2_, 200 μM PbCl_2_ and recombinant *D. radiodurans* strain LOPM.

Exposure to a solution containing 100 µM CdCl_2_ and 200 µM PbCl_2_ (Cd and Pb) without bacteria induced severe toxicity in rice plants. After Cd and Pb exposure, the plant height and root length after ten days treatment were only about 53.8% and 54.3% those of control, respectively (Figure 3B,C). The dry biomass of the plant was only about 54.8% of control (Figure 3D).

Rice seedling growth showed improvement in the presence of *D. radiodurans* in the supplemented medium, and the recombinant strain LOPM showed a better effect than that of the wild-type strain R1 (Figure 3A), indicating the recombinant strain LOPM cells could efficiently mitigate Cd and Pb toxicity in seedlings under Cd and Pb stress. Following Cd and Pb treatment, the plant height and root length treated by recombinant strain LOPM were about 90.8% and 90.5% of those of the control, respectively (Figure 3B,C). Meanwhile, the dry biomass of plants cultured with recombinant strain LOPM restored 90.5% of the untreated plants, 1.65-fold higher than that of Cd- and Pb-treated plants (Figure 3D). All these data suggested that recombinant strain LOPM exhibited a superior ability to reduce Cd and Pb toxicity in rice.

### 2.4. Root Colonization of D. radioduran Recombinant Strain LOPM

**Figure 4:**
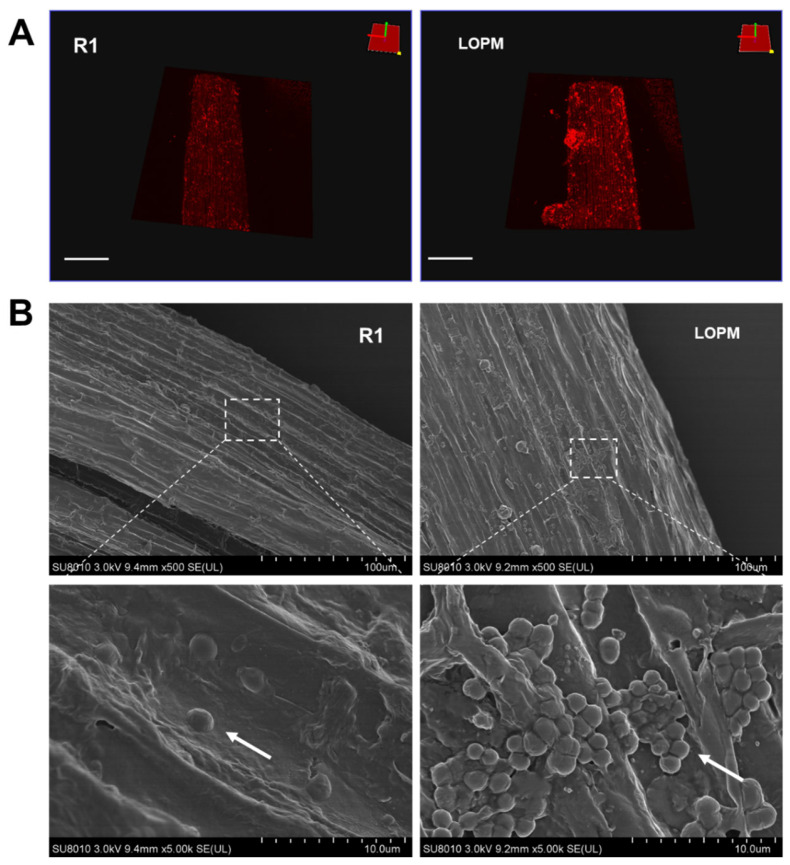
Fluorescence labeling (**A**) and SEM (**B**) analysis on root colonization of *D. radiodurans* wild-type strain R1 and recombinant strain LOPM. The rice root tissue co-cultured with colonized bacterial cells was observed by laser scanning confocal microscopy. The bacterial cell membrane was stained by Dil (red fluorescence). The colonized bacteria cells on the roots were indicated by the white arrows, scale bar = 1 mm.

Root colonization can indicate the existence of interactions between plants and microorganisms. Many microorganisms colonized on root could effectively enhance the tolerance of plants under heavy metal stress through the active plant–microorganism interaction [32,33]. Fluorescence 3D images obtained by laser scanning confocal microscopy clearly revealed that much more recombinant strain LOPM cells were colonized on the surface of the root, compared with the wild-type strain R1 (Figure 4A,B). Moreover, SEM images showed that recombinant strain LOPM cells accumulate on the root surface (Figure 4B), while cells of the wild-type strain R1 distributed scattered on the root surface.

### 2.5. D. radioduran Recombinant Strain LOPM Reduced Cd and Pb Accumulation in Rice

**Figure 5:**
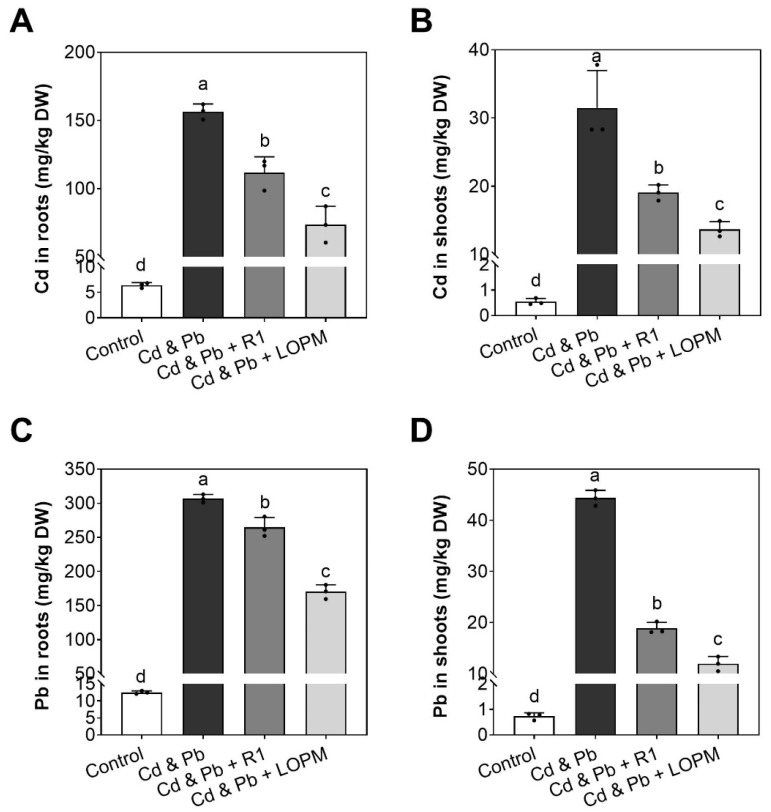
The content of cadmium (Cd) and lead (Pb) in the roots and shoots of rice plants after 10 days of growth with different treatments (**A**–**D**). The analyses were performed with three replications. The significant difference (*p* ˂ 0.05) was indicated by the different letters above the error bars. The details of different supplements were illustrated in Figure 3.

Without the Cd and Pb exposure, the cultured rice seedlings exhibited low concentrations of Cd or Pb in the roots (6.4 mg/kg DW Cd, 12.5 mg/kg DW Pb) and shoots (0.54 mg/kg DW Cd, 0.73 mg/kg DW Pb), respectively (Figure 5A–D). Exposure by Cd and Pb, the contents of Cd and Pb were markedly elevated in both root and shoot. After ten days of growth in a Cd- and Pb-supplemented medium, the Cd content of seedlings reached 156.4 (in root) and 31.5 mg/kg (in shoot), which were 24.4- and 58.0-fold higher than those in the control, respectively (Figure 5A,B).

Comparing to the plants singly treated with Cd and Pb, the wild-type strain R1 cells cultured with rice could notably decrease the Cd level to 71.4% (in root) and 60.5% (in shoot), and reduce the Pb level to 86.2% (in root) and 42.5% (in shoot), respectively (Figure 5A–D). Moreover, the recombinant strain LOPM cells reduced the Cd level to 47.0% (in root) and 43.4% (in shoot), and reduced the Pb level to 55.4% (in root) and 26.9% (in shoot), respectively, as compared to the plants treated with Cd and Pb (Figure 5A–D). All these data indicated that recombinant strain LOPM cells are more effective at preventing the uptake of Cd and Pb by rice, as compared with the wild-type strain R1.

### 2.6. D. radioduran Recombinant Strain LOPM Alleviated Cd- and Pb-Induced Oxidative Stress in Rice

In order to study how recombinant strain LOPM efficiently protected rice from Cd and Pb, we quantified the contents of oxygen species (ROS), malondialdehyde (MDA), proline (Pro) and activities of three antioxidant enzymes (CAT, POD and SOD) in rice seedlings cultured with wild-type strain R1 and recombinant strain LOPM cells under Cd and Pb stress.

**Figure 6:**
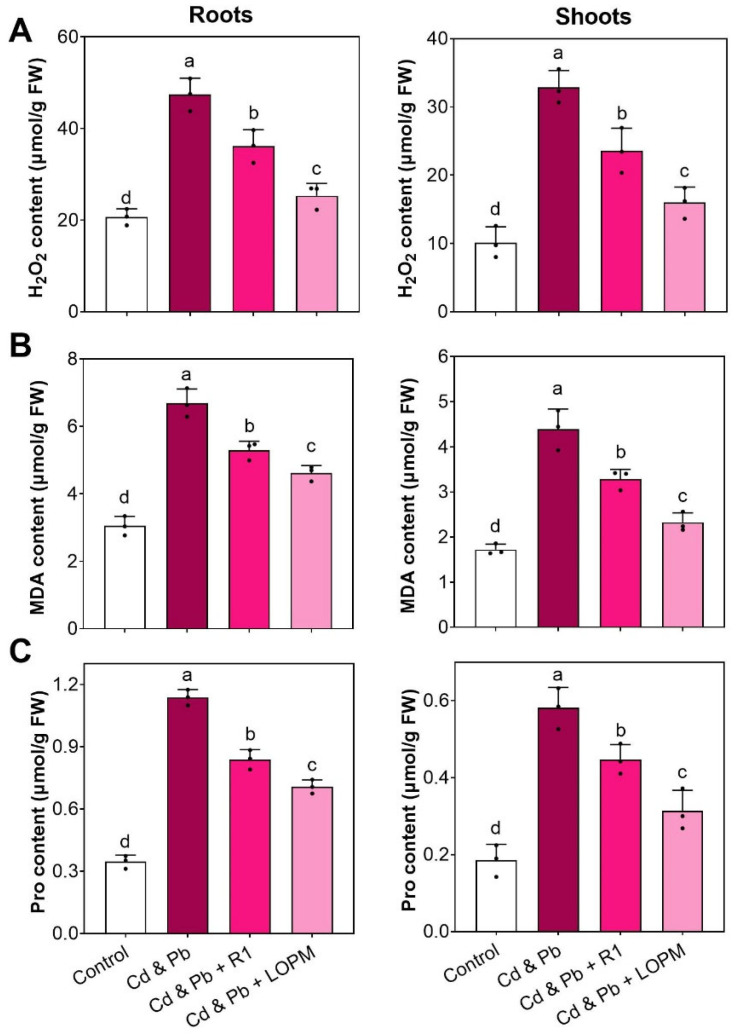
Accumulation of H_2_O_2_, malondialdehyde (MDA), and proline (Pro) in rice plants after 10 days of growth with different treatments. The accumulations of H_2_O_2_ (**A**), MDA (**B**), and Pro (**C**) in roots and leaves. The significant difference (*p* < 0.05) was indicated by the different letters above the error bars. The details of different supplements were illustrated in Figure 3.

Cd and Pb exposure induced oxidative stress by rising ROS levels, including H_2_O_2_ (Figure 6A), which are toxic to plant tissues and cells. Cd and Pb exposure for ten days strongly increased the H_2_O_2_ contents by 2.29-fold in root and 3.24-fold in shoot, respectively. Compared to rice treated with Cd and Pb, the recombinant strain LOPM supplements lowered H_2_O_2_ levels by 46.6% in the root and by 51.3% in the shoot, respectively (Figure 6A). Moreover, ROS induced by Cd and Pb could cause malondialdehyde (MDA) accumulation. However, the MDA level was notably reduced in rice when it was cultured with recombinant strain LOPM cells (Figure 6B).

Proline (Pro), as a stress-related FAA in plants, could be regarded as an indicator of oxidative pressure [34]. With Cd and Pb treatment, the amounts of Pro were elevated by 3.28-fold in root and 3.14-fold in shoot, respectively (Figure 6C). However, recombinant strain LOPM treatment markedly reduced the amounts of Pro by 37.8% in root and 46.1% in shoot under Cd and Pb stress (Figure 6C).

**Figure 7:**
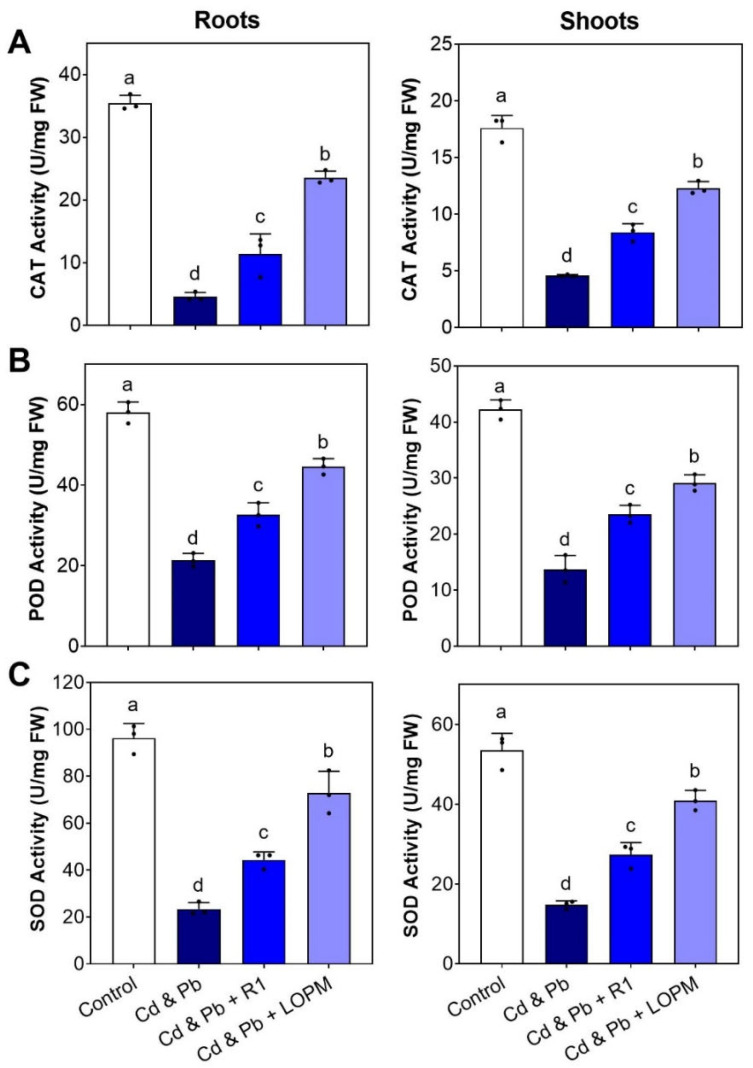
CAT, POD, and SOD activities in rice plants after 10 days of growth with different treatments. The CAT (**A**), POD (**B**), and SOD (**C**) activities were detected in roots and leaves. The significant difference (*p* ˂ 0.05) was indicated by the different letters above the error bars. The details of different supplements were illustrated in Figure 3.

Cd and Pb exposure for ten days reduced the activities of the three antioxidant enzymes (CAT, POD and SOD) in rice by 87.1%, 63.1%, and 75.7% in root, and 73.9%, 67.5%, and 72.3% in shoot, respectively (Figure 7A–C). These findings corroborated earlier studies which reported that heavy metal treatment reduced the activities of these antioxidant enzymes [35]. Adding recombinant strain LOPM cells significantly enhanced the CAT activity by 5.16-fold (root) and 2.68-fold (shoot); POD activity by 2.09-fold (root) and 2.12-fold (shoot), SOD activity by 3.12-fold (root) and 2.76-fold (shoot), respectively, compared to the rice with Cd and Pb treatment (Figure 7A–C). Previous studies also showed that a metal-resistant bacteria could reduce cadmium toxicity and regulate antioxidant machinery in rice [36]. Moreover, the recombinant strain LOPM cells supplement exhibited a great performance for enhancing the activities of CAT, POD and SOD in rice than that of wild-type strain R1. These results showed that the recombinant strain LOPM, could effectively reduce Cd and Pb toxicity via scavenging ROS and enhancing the activities of antioxidant enzymes in rice.

## 3. Discussion

This study illustrated that surface display of multiple metal-binding domains in *Deinococcus radiodurans* could efficiently mitigate Cd and Pb toxicity in rice seedlings. Both Cd and Pb accumulation could generate ROS, thus inducing oxidative stress in plants [3,5], and the oxidative stress may lead to a series of harmful effects on plants including DNA damage, protein oxidation, lipid peroxidation, and reduced photosynthesis [2]. Moreover, the accumulation of Cd and Pb in rice grains may cause damage to human body via the food chains [3,5]. Under Cd and Pb stress, rice seedlings displayed a significant decrease in plant height, root length, and dry biomass (Figure 3A–D). While cultured with *D. radiodurans* wild-type strain R1 or recombinant strain LOPM cells rice seedling’s growth and phenotypes were substantially restored (Figure 3A–D). The recombinant strain LOPM showed a significantly higher accumulation of cells on the root surface compared to the wild-type strain R1 (Figure 4A,B), possibly due to the stronger tolerance and better growth under Cd and Pb exposure.

OmpA is a protein located in the outer membrane of Gram-negative bacteria [37]. This protein is engaged in a variety of functions such as adhesion, toxicity, and biofilm formation [37]. Some functions, such as adhesion and biofilm formation, may also enhance heavy metal absorption. As an efficient display system, Lpp-OmpA could transfer many types of proteins onto the bacterial surface [23,24,25,26,27]. By expressing metallothionein fused with Lpp-OmpA, the recombinant *E. coli* displayed a 4.9-fold greater Cd adsorption compared to wild-type [26]. The fusion of PbrR with Lpp-OmpA in *E. coli* also exhibited a significantly improved capacity for Pb adsorption [27]. Exposure by Cd and Pb simultaneously, the fusion of Lpp-OmpA with MTT5 and PbBD in *D. radiodurans* exhibited significantly enhanced capacity for Cd and Pb removal (Figure 2A–D). For other heavy metals, such as Hg, Cu, and Cr, the Lpp-OmpA module may also be fused with the specific metal-binding protein to apply for the bacterial adsorption of heavy metals in future research. Moreover, metal-binding proteins could be connected together for simultaneous adsorption of multiple heavy metals.

Natural water bodies and soils can be contaminated with multiple heavy metals. In response to the heavy metals, plants growing in these places could show browning of roots, chlorosis, stunted growth, and even death. And multiple heavy metals may impede many metabolic processes essential for plant survival and growth. The accumulation of heavy metals in soil and plants can further pose significant risks to human health. Monitoring and controlling these hazardous pollutants are of great urgency. Directly collecting soil and plant samples from areas of concern could be a simple method of monitoring the heavy metal, while microorganisms with genetic engineering modification may be used as bioindicators to assess heavy metal contamination. For controlling heavy metals, bacteria capable of simultaneously processing multiple heavy metals may be an effective bioremediation technology. *D. radiodurans* has been proved to have a high removal efficiency for Cd (78%) and Pb (86%) under the single heavy metal treatment [31]. Even with exposure by Cd and Pb simultaneously, the wild-type strain R1 strain still exhibits an effective removal efficiency for Cd (72.3%) and Pb (82.5%) (Figure 2H). This indicated that *D. radiodurans* may adapt to a more complicated environment with multiple heavy metals. Previous study also showed that the S-layer mutant strain (*Δdr2577*) exhibits a higher removal efficiency for Cd (88.2%) and Pb^2+^ (93%) [31]. While the recombinant strain LOPM even could remove 84.9% Cd and 94.1% Pb under the condition of simultaneous existence of Cd and Pb (Figure 2H).

*D. radiodurans* possesses a rich array of antioxidants, such as catalases, superoxide dismutases, and small molecule antioxidants, with a notable abundance of Mn^2+^metabolite complexes [38,39]. Previous study reported that *D. radiodurans* colonized on the surface of root could release Mn^2+^-metabolite and glutamate to suppress heavy metal absorption and heavy metal-induced oxidative stress [31]. And the mutation of S-layer protein on the cell envelope enhances the release of Mn^2+^-metabolite and glutamate [31]. Our study revealed that the recombinant LOPM strain, as a supplement, outperforms the wild-type strain R1 strain in boosting the activities of antioxidant enzymes in rice exposed to Cd and Pb stress (Figure 7A–C). The expression of LOPM on the cell envelops of *D. radiodurans* may influence the release of these metabolites; thus, the LOPM cells accumulated on the surface of root may enhance the activities of the antioxidant enzymes in plant resistance through the release of more metabolites. Exploring the interaction between genetically modified *D. radiodurans* and rice contaminated with Cd/Pb is worthy of investigation.

## 4. Materials and Methods

### 4.1. Bacterial Strains and Culture Media

The plasmids and strains used in this study are listed in Table S1. *Deinococcus radiodurans* strains were grown at 30 °C in TGY medium (0.5% tryptone, 0.1% glucose, and 0.3% yeast extract) or on TGY plates added with 1.5% Bacto-agar. *Escherichia coli* and *Cupriavidus metallidurans* were grown in Luria-Bertani (LB) broth or LB agar at 37 °C.

### 4.2. Construction of Recombined Strain LOPM

The genomic DNA from *Escherichia coli* K12 and *Cupriavidus metallidurans* (DSM 2839) were extracted using the bacterial genomic DNA extraction kit (Tiangen Biochemical, Beijing, China). The LPP-OmpA and PbBD DNA fragments were cloned using these DNA templates. The MTT5 gene fragment was obtained by gene synthesis (Tsingke Biotech, Hangzhou, China). The upstream primer LPP-OmpA-F and the downstream primer LPP-OmpA-R were used to amplify the LPP-OmpA fragment. The upstream primer PbBD-F and the downstream primer PbBD-R were used to amplify the PbBD fragment. The upstream primer MTT5-F and MTT5-R were used to amplify the MTT5 fragment. All lowercase letters are flexible linkers and homology arms sequences that connect the upstream and downstream fragments. These three DNA fragments were connected to the deinococcal over-expression plasmid pRAD-P8 to construct the P8-LPP-OmpA-PbBD-MTT5 plasmid. The recombinant plasmid pRAD-P8-LPP-OmpA-PbBD-MTT5 was transferred into *D. radiodurans* R1, then screened with a TGY-resistant medium (0.5% trypton, 0.1% glucose, and 0.3% yeast extract) containing 3.4 μg/mL chloramphenicol and incubated at 30 °C for 4–5 days to obtain the recombinant strain LOPM. The bacteria strains, plasmids, and primers used in this study were listed in Tables S1 and S2.

### 4.3. Plant Materials and Treatments

The sterilization process of rice seeds (*Oryza sativa* ssp *Japonica*) involved a 20 min soak in a 2% (*w*/*v*) NaClO solution, followed by three rinses with ultrapure water and a 24 h immersion in ultrapure water at 30 °C. Subsequently, these seeds were transferred to 96-well hydroponic boxes filled with half-strength Murashige–Skoog (MS) liquid medium. The boxes were then placed in a growth chamber (1 L volume) with conditions set at 75% humidity, a light cycle of 16 h at 30 °C, and a dark cycle of 18 h at 25 °C. For the heavy metal treatment, 14-day-old rice seedlings were cultivated in ½ MS liquid medium supplemented with 100 μM CdCl_2_ and 200 μM PbCl_2_, with or without the addition of bacteria (OD_600_ = 1.0) harvested by centrifugation from a 500 mL TGY broth culture. After 10 days of treatment, the rice seedlings were sampled for molecular, physiological, and phenotypic analysis. This entire process was replicated three times.

### 4.4. Transmission Electron Microscopy (TEM) and Scanning Electron Microscopy (SEM)

For scanning electron microscopy (SEM) analysis, both the wild-type *D. radiodurans* R1 and the recombinant LOPM strains were grown in TGY broth at 30 °C until they reached an OD_600_ of 1.0. Following centrifugation, the cells were resuspended in 100 µM CdCl_2_, 200 µM PbCl_2_, and a combination of 100 µM CdCl_2_ and 200 µM PbCl_2_, respectively. After a 4 h incubation, the bacteria were collected by centrifugation. Approximately 300 mg of bacterial or plant tissue samples were then processed using the previously described method [40]. The samples were fixed with 2.5% glutaraldehyde in phosphate buffer (pH 7.0) overnight, followed by dehydration and coating with a gold–palladium layer before SEM (Hitachi, Tokyo, Japan) observation.

For transmission electron microscopy (TEM) analysis, the fixation and dehydration procedures were the same as above, with the addition of an agar-embedding step. The samples were sectioned thinly, stained with uranyl acetate for 15 min, and then examined under TEM (Hitachi, Tokyo, Japan).

### 4.5. Assessment of Bacterial Colonization in Rice

The bacterial colonization in rice was assessed using a previously described method [31].

### 4.6. Metal Ion Detection

The contents of metal ions in bacteria and plants were quantified using a previously described method [31,41].

### 4.7. Determination of Hydrogen Peroxide (H_2_O_2_) Levels

The content of hydrogen peroxide (H_2_O_2_) was quantified using the procedure outlined in a previous study [42].

### 4.8. Determination of Malondialdehyde (MDA) Levels

The MDA accumulation was assessed according to the method described [31]. Roughly 100 mg of rice tissue was blended with 15 mL of 10% trichloroacetic acid, followed by centrifugation at 12,000× *g* for 20 min. The resulting supernatant was combined with an equal volume of thiobarbituric acid and incubated at 95 °C for 30 min before being chilled on ice. After a subsequent centrifugation at 12,000× *g* for 20 min, the absorbance of the supernatant was recorded at 450, 532, and 600 nm, respectively.

### 4.9. Measurements of Antioxidant Enzyme Activities

The activities of antioxidant enzymes in rice, such as superoxide dismutase (SOD), peroxidase (POD), and catalase (CAT), were measured using the procedures outlined in previous studies [43,44,45].

### 4.10. Statistical Analysis

To determine the statistical significance of the results, Student’s *t*-tests were applied, and a *p*-value below 0.05 was taken as indicating significance. The dataset is presented as mean values accompanied by standard deviations, based on at least three independent experiments (mean ± SD). Bars in the figures with different letters denote statistically significant differences.

## 5. Conclusions

In conclusion, surface display of multiple metal-binding domains in *Deinococcus radiodurans* could enhance its Cd and Pb tolerance and adsorption. And the Cd and Pb tolerance recombinant strain LOPM displayed an enhanced root colonization on root of rice, efficiently chelating Cd and Pb and promoting enzymatic antioxidants of rice, thus enhancing the resistance of rice to Cd and Pb stress. Our results demonstrated that the surface display of multiple metal-binding domains in *D. radiodurans* significantly decreases the harmful effects of Cd and Pb on rice. Managing cadmium and lead pollution by surface display of multiple metal-binding domains in bacteria may provide an effective method to reduce multi-heavy metal contamination and stress in plants. Additionally, the utility of extreme microorganism *D. radiodurans* in bioremediation applications deserves further exploration.

## Data Availability

The raw data supporting the conclusions of this article will be made available by the authors without undue reservation.

## Supplementary Materials

The following supporting information can be downloaded: https://www.mdpi.com/article/10.3390/ijms252312570/s1.
