# Supplementary material for: Surface Display of Multiple Metal-Binding Domains in Deinococcus radiodurans Alleviates Cadmium and Lead Toxicity in Rice
Source: Int J Mol Sci. 2024 Nov 22;25(23):12570. doi: 10.3390/ijms252312570 (PMC11640945; doi:10.3390/ijms252312570)
Supplement: Supplementary file 1 [file ijms-25-12570-s001.zip › ijms-3310953-supplementary.pdf]

Supplementary materials

Surface display of multiple metal binding domains in *Deinococcus radiodurans* alleviates cadmium and lead toxicity in rice

Liangyan Wang[1], Yudong Wang[1], Shang Dai[1,*], Binqiang Wang[1, 2,*]

1 Institute of Biophysics, College of Life Sciences, Zhejiang University, Hangzhou, 310029, China.

2 State Key Laboratory of Clean Energy Utilization, Zhejiang University, Hangzhou, 310029, China.

* Correspondence: wangbinqiang@zju.edu.cn; daishang@zju.edu.cn

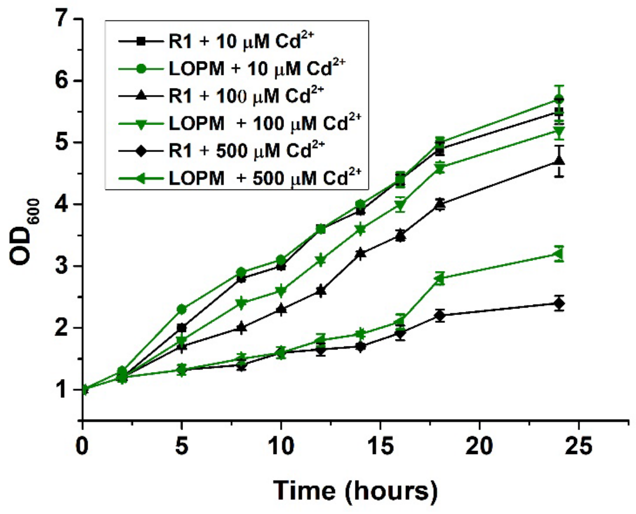

Figure S1. Effect of Cd on the growth of *D. radiodurans* wild-type R1 and recombinant strains LOPM. Growth curves of bacteria under the treatments of different concentration of Cd.

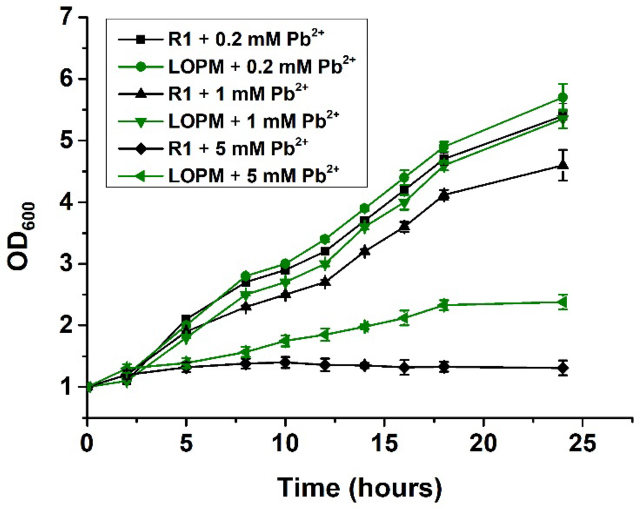

Figure S2. Effect of Pb on the growth of *D. radiodurans* wild-type R1 and recombinant strains LOPM. Growth curves of bacteria under the treatments of different concentration of Pb.

Table S1. Strains and plasmids used in this study.

Strains or plasmids	Description	Source
**Strains**		
D. radiodurans R1	ATCC 13939	Lab stock
D. radiodurans LOPM	*D. radiodurans R1* inserted with plasmid pRAD-P8-LPP-OmpA-PbBD-MTT5	This study
Escherichia coli	Strain K12	Lab stock
Cupriavidus metallidurans	DSM 2839	Lab stock
**Plasmid**		
pRAD	pRADZ3 derivative in which lacZ is replacedwith the kanamycin gene (AprKmrCmr)	Lab stock

Table S2. Primers used in this study.

Primer	Sequence (5'- 3')
Protein expression	
LPP-OmpA-F	gattatcactattctgATGAAAGCCACCAAACTGGT
LPP-OmpA-R	gctgccgccccACGCGTTGCAATTTCCGG
PbBD-F	ggcggcggcggcagcCAGTTCATTCGTCACTGCCGG
PbBD-R	gctgccgccgccgccgctgccgccgccgccCGACAGTCCCTGCAGAATCC
MTT5-F	ggcggcggcggcagcggcggcggcggcagcGATAAAAATTTCTGGTGAAAGCACT
MTT5-R	cctgcaggtcgaatcggatccTCAGCAACTACCTCCAGG

All lowercase letters are flexible linkers and homology arms sequences that connect the upstream and downstream fragments.
